# A Dosimetric Study of Using Fixed-Jaw Volumetric Modulated Arc Therapy for the Treatment of Nasopharyngeal Carcinoma with Cervical Lymph Node Metastasis

**DOI:** 10.1371/journal.pone.0156675

**Published:** 2016-05-27

**Authors:** Wu-Zhe Zhang, Jia-Yang Lu, Jian-Zhou Chen, Tian-Tian Zhai, Bao-Tian Huang, De-Rui Li, Chuang-Zhen Chen

**Affiliations:** Department of Radiation Oncology, Cancer Hospital of Shantou University Medical College, Shantou, Guangdong, China; ENEA, ITALY

## Abstract

**Purpose:**

To study the dosimetric difference between fixed-jaw volumetric modulated radiotherapy (FJ-VMAT) and large-field volumetric modulated radiotherapy (LF-VMAT) for nasopharyngeal carcinoma (NPC) with cervical lymph node metastasis.

**Methods:**

Computed tomography (CT) datasets of 10 NPC patients undergoing chemoradiotherapy were used to generate LF-VMAT and FJ-VMAT plans in the Eclipse version 10.0 treatment planning system. These two kinds of plans were then compared with respect to planning-target-volume (PTV) coverage, conformity index (CI), homogeneity index (HI), organ-at-risk sparing, monitor units (MUs) and treatment time (TT).

**Results:**

The FJ-VMAT plans provided lower D_2%_ of PGTVnd (PTV of lymph nodes), PTV1 (high-risk PTV) and PTV2 (low-risk PTV) than did the LF-VMAT plans, whereas no significant differences were observed in PGTVnx (PTV of primary nasopharyngeal tumor). The FJ-VMAT plans provided lower doses delivered to the planning organ at risk (OAR) volumes (PRVs) of both brainstem and spinal cord, both parotid glands and normal tissue than did the LF-VMAT plans, whereas no significant differences were observed with respect to the oral cavity and larynx. The MUs of the FJ-VMAT plans (683 ± 87) were increased by 22% ± 12% compared with the LF-VMAT plans (559 ± 62). In terms of the TT, no significant difference was found between the two kinds of plans.

**Conclusions:**

FJ-VMAT was similar or slightly superior to LF-VMAT in terms of PTV coverage and was significantly superior in terms of OAR sparing, at the expense of increased MUs.

## Introduction

Radiotherapy is the main treatment paradigm for nasopharyngeal carcinoma (NPC). The radiotherapy volumes are complex due to the location of the tumors adjacent to organs at risk (OARs) and the specific distributions of lymph node drainage area. With the development of radiotherapy techniques, intensity-modulated radiotherapy (IMRT) and volumetric modulated radiotherapy (VMAT) have been widely used in the treatment of NPC [1–3]. VMAT is an extension of IMRT with a continually changing multi-leaf collimator (MLC) aperture and a continually rotational gantry; furthermore, VMAT allows for the beam intensity to be adjusted at various gantry angles to deliver specific doses to targets and spare OARs. Compared with the static-gantry IMRT technique, VMAT can achieve similar or superior dosimetric results, with reduced treatment time and improved treatment efficiency [4,5]. The planning target volumes (PTVs) of NPC are typically large in scale, with irregular shapes. In conventional VMAT, the field size is typically 20–25 cm along the transverse direction to cover the entire PTV region (large-field VMAT, LF-VMAT). Unfortunately, the MLC in Varian linear accelerators suffers from a mechanical limitation because the maximum leaf span of the MLC is 15 cm. Accordingly, if the X-direction jaw width is > 15 cm, the MLC modulation level would be reduced, thus resulting in relatively unsatisfactory target dose distribution and OAR sparing. To achieve higher modulation, the X-direction jaws should be set to ≤ 15 cm. As demonstrated by past studies [6,7], compared with split-field IMRT, fixed-jaw IMRT provides better logistics, lower monitor units (MUs) and shorter treatment times, but both techniques involve longer treatment times and a greater number of MUs than those required by VMAT. Our previous study [8] demonstrated that fixed-jaw VMAT (FJ-VMAT) has certain dosimetric advantages in treating cervical cancer, but to date, whether FJ-VMAT can provide dosimetric benefits for NPC is still unknown. Therefore, we compared FJ-VMAT with the conventional LF-VMAT for NPC with the aim of providing a theoretical basis for choosing a reasonable radiotherapy technique.

## Material and Methods

### Ethics Statement

The study protocol was approved by the Ethical Commission of Cancer Hospital of Shantou University Medical College. Because this study was not treatment-based, our institutional review board waived the need for written informed consent from the participants. Patient information was anonymized to protect patient confidentiality.

### Patients

The computer tomography (CT) datasets of 10 patients with pathologically confirmed nasopharyngeal squamous cell carcinoma without distant metastasis, treated with chemoradiation at the Cancer Hospital of Shantou University Medical College from January 2013 to June 2014, were identified. The patients (median age 45 years, age range 23–68 years) included 6 females and 4 males. According to the criteria outlined in the seventh edition of the American Joint Committee on Cancer (AJCC) manual (2010), the tumors were in stages T2–T4, N1–N3 and M0, as shown in Table 1. The maximum PTV width was 21.8–25.3 cm and was thus beyond the MLC leaf span. Treatments were planned with patients in the supine position with arms resting at both sides of the body under head-neck-shoulder thermoplastic mask immobilization. The CT datasets, with 3-mm-thick slices gathered from the head to 2 cm below the sternoclavicular joint, were acquired using a 16-slice CT scanner (Philips Brilliance CT Big Bore Oncology Configuration, Cleveland, OH, United States) for all patients. The CT images were transferred to the Eclipse version 10.0 treatment planning system (Varian Medical Systems, Palo Alto, CA, United States) for target-volume and OAR contouring and subsequent treatment planning.

**Table 1:** Tumor characteristics.

No.	Stage	Volume of GTVnx (cm^3^)	Volume of GTVnd (cm^3^)	Volume of PGTVnx (cm^3^)	Volume of PGTVnd (cm^3^)	Volume of PTV1 (cm^3^)	Volume of PTV2 (cm^3^)	Maximum width of PTV (cm)
**1**	T_3_N_2_M_0_	30.00	2.09	66.16	18.90	468.60	194.03	24.60
**2**	T_2_N_3_M_0_	15.52	41.69	41.66	116.33	648.89	121.90	22.80
**3**	T_2_N_2_M_0_	13.49	17.89	45.89	110.11	504.94	180.86	25.00
**4**	T_3_N_2_M_0_	37.69	4.65	73.36	35.28	386.80	199.22	23.20
**5**	T_2_N_2_M_0_	10.94	13.42	33.74	50.76	443.72	142.58	25.30
**6**	T_3_N_3_M_0_	75.02	29.61	134.03	100.60	732.75	154.66	23.80
**7**	T_2_N_1_M_0_	4.37	1.68	20.30	10.50	359.87	81.37	21.80
**8**	T_2_N_2_M_0_	7.99	11.92	28.55	49.96	511.97	271.42	22.70
**9**	T_2_N_1_M_0_	23.00	3.45	47.16	12.24	556.12	59.46	23.00
**10**	T_4_N_1_M_0_	52.84	7.26	99.44	30.64	529.80	53.37	22.90

GTVnx, gross tumor volume of primary nasopharynx; GTVnd, gross tumor volume of positive lymph nodes; PTV, planning target volume; PGTVnx, PTV of GTVnx; PGTVnd, PTV of GTVnd; PTV1, high-risk PTV; PTV2, low-risk PTV.

### Target/OAR delineation

The attending radiation oncologists for each patient contoured the primary nasopharyngeal gross tumor volume (GTVnx), involved lymph nodes (GTVnd), clinical target volumes (CTVs), PTVs and OARs in Eclipse. GTVnx and GTVnd were determined using the planning CT, magnetic resonance imaging (MRI), positron emission tomography (PET) and clinical information. CTV1 was defined as the GTVnx and GTVnd plus a 5- to 10-mm margin for potential microscopic spread. When a positive lymph node was found in the lower neck (level IV and/or Vb), CTV1 generally included the ipsilateral region of the retropharyngeal lymph node and level II, III, IV and V. When a positive lymph node was found in only the upper neck (level II, III and Va), CTV1 included ipsilateral level II, III and Va while the low-risk CTV (CTV2) included ipsilateral level IV and Vb. For patients with unilateral neck lymph node metastasis, level II, III and Va of contralateral neck were included in CTV1. The four PTVs, PGTVnx, PGTVnd, PTV1 and PTV2, were generated by adding 5-mm outer margins to GTVnx, GTVnd, CTV1, CTV2, respectively. The OARs included spinal cord (SC), brainstem (Bstem), parotid, oral cavity, and larynx. The planning OAR volumes (PRVs) of the spinal cord and brainstem, which were generated with 5- and 3-mm outer margins from the SC and Bstem, were denoted as PRV_SC_ and PRV_Bstem_, respectively. Surrounding normal tissue region was defined as the body minus all of the PTVs and was denoted as B-P.

### Treatment planning techniques and planning objectives

For each case, the LF-VMAT and FJ-VMAT plans were generated in Eclipse using 6-MV X-ray beams with a maximum dose rate of 600 MU/min from the TrueBeam linear accelerator (Varian Medical Systems, Palo Alto, CA, United States). The maximum speed of gantry rotation was 6°/s. Both the dose rate and gantry speed are variable in VMAT technique. The leaf widths of the MLC were 0.5 and 1 cm for the central and outer 20 cm area, respectively. Both the LF-VMAT and FJ-VMAT plans used coplanar dual arcs (clockwise rotation from 181° to 179° and counter-clockwise rotation from 179° to 181°) with couch 0° and with collimator rotated to 15°/345° to minimize the contribution of the tongue-and-groove effect to the dose. In the LF-VMAT plans, the jaw width was automatically set to cover the entire target area. In the FJ-VMAT plans, the X-direction jaw width was set to 15 cm. The simultaneous integrated boost technique was used. PTV prescription doses were as follows: PGTVnx 70.4 Gy, PGTVnd 68 Gy, PTV1 60 Gy, and PTV2 54 Gy (32 fractions). The two types of plans were optimized using the same planning objective settings. The planning objective for the PGTVnx was to achieve at least 95% of the PTV volume that received 100% of the prescribed dose, with the maximum dose < 77.44 Gy. The dose constraints for the OARs were set as follows (D_x%_ represents the dose reached or exceeded in x% of the volume and V_x_ represents the volume receiving at least x dose): the D_max_ (maximum dose) of PRVsc < 45 Gy or the V_50Gy_ of PRVsc < 1 cc; the D_max_ of PRV_Bstem_ < 54 Gy or the V_60Gy_ of PRV_Bstem_ < 1 cc; the V_30Gy_ of parotid < 50%; and the D_mean_ of oral cavity or larynx < 40 Gy. Fulfilling the objectives for the PTV was prioritized above fulfilling those for the OARs. To ensure consistency among the planning techniques, physicists with over 9 years of experience in IMRT and over 4 years of experience in VMAT planning designed all treatment plans. The planning requirements and techniques for planners were also aligned using standard protocols and departmental procedures. Final doses were calculated using the Anisotropic Analytical Algorithm (AAA), version 10.0.28, with a 2.5-mm grid size.

### Plan evaluation

Quantitative evaluation of the plans was performed using the statistical data from the cumulative dose-volume histogram (DVH). According to the International Commission on Radiation Units and Measurements (ICRU) report 83 [9], for the PTV, D_98%_ and D_2%_ were chosen as near-minimal and near-maximal doses, respectively. The homogeneity index (HI), which was used to evaluate the target dose homogeneity, was defined as follows:

$$HI=\frac{D_{2\%}-D_{98\%}}{D_{50\%}}$$

The conformity index (CI), which was used to evaluate target dose conformity, was defined [10] as follows:

$$CI=\frac{{\left(V_{T,ref}\right)}^{2}}{V_{T}\times V_{ref}}$$

Where V_T,ref_ represents the volume of the target receiving a dose ≥ the reference dose, V_T_ represents the physical volume of the target, and V_ref_ represents the total tissue volume receiving a dose ≥ the reference dose. The reference dose was the prescribed dose for the target in this study. A CI value of 1 represents the ideal level of conformity, whereas an HI value of 0 represents the ideal level of homogeneity.

The evaluation indicators for OARs were set as follows: (1) D_max_, V_45Gy_ and V_50Gy_ for PRVsc; (2) D_max_, V_54Gy_ and V_60Gy_ for PRV_Bstem_; (3) D_mean_, V_30Gy_ for parotid; and (4) V_5Gy_, V_10Gy_, V_15Gy_, V_20Gy_ and V_30Gy_ for B-P. The delivery parameters included the following: (1) MU and (2) treatment time (TT).

### Statistical analysis

The Statistical Package for Social Sciences (SPSS, Inc., Chicago, IL, USA) was used for the statistical analyses in this study. Comparisons of the dose-volume data were performed using a paired, two-tailed Student’s t-test. When the data did not follow a normal distribution, a Wilcoxon matched-pair signed-rank test was used. The results were considered to be statistically significant for *P*-values of < 0.05.

## Results

### Target coverage, homogeneity, and conformity

**Fig 1:**
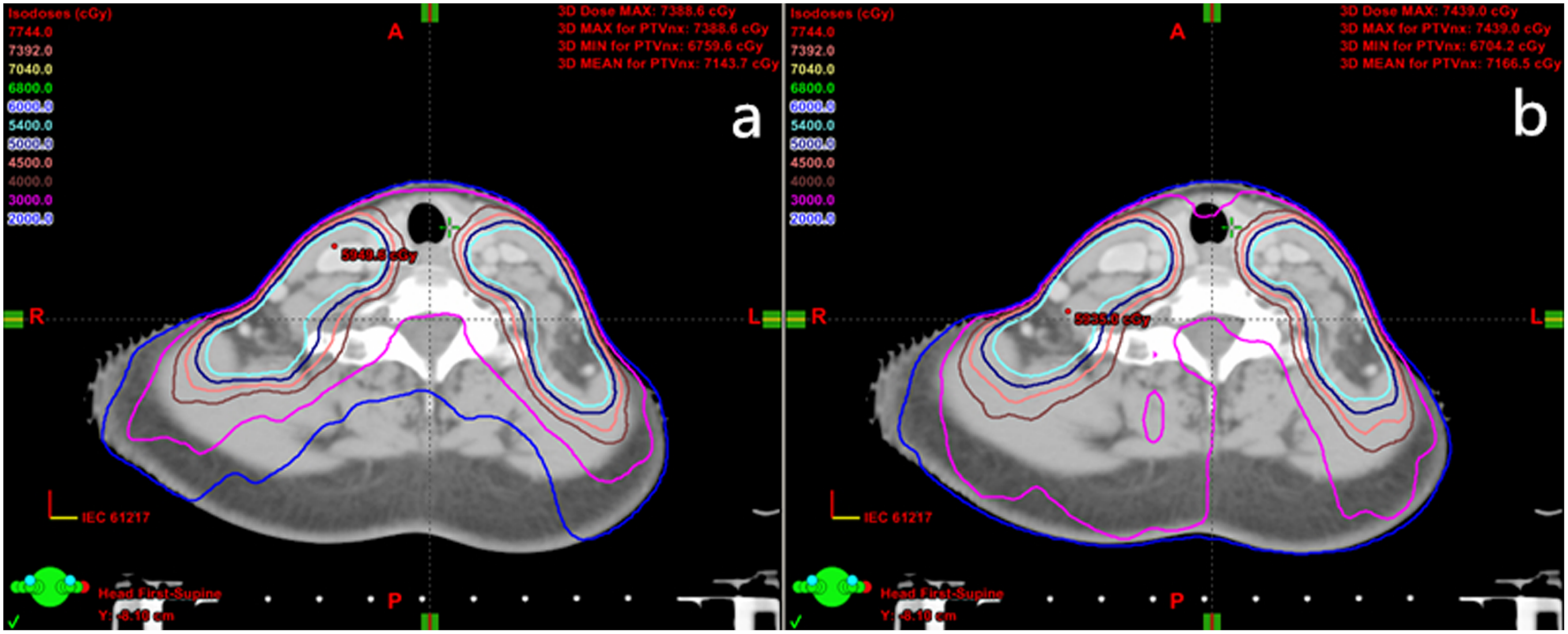
Isodose distributions of the large-field volumetric arc therapy (LF-VMAT) and fixed-jaw VMAT (FJ-VMAT) plans. (a) The FJ-VMAT plans. (b) The LF-VMAT plans. Eleven dose lines of 20–77 Gy from the outside to the inside of each CT image.

**Fig 2:**
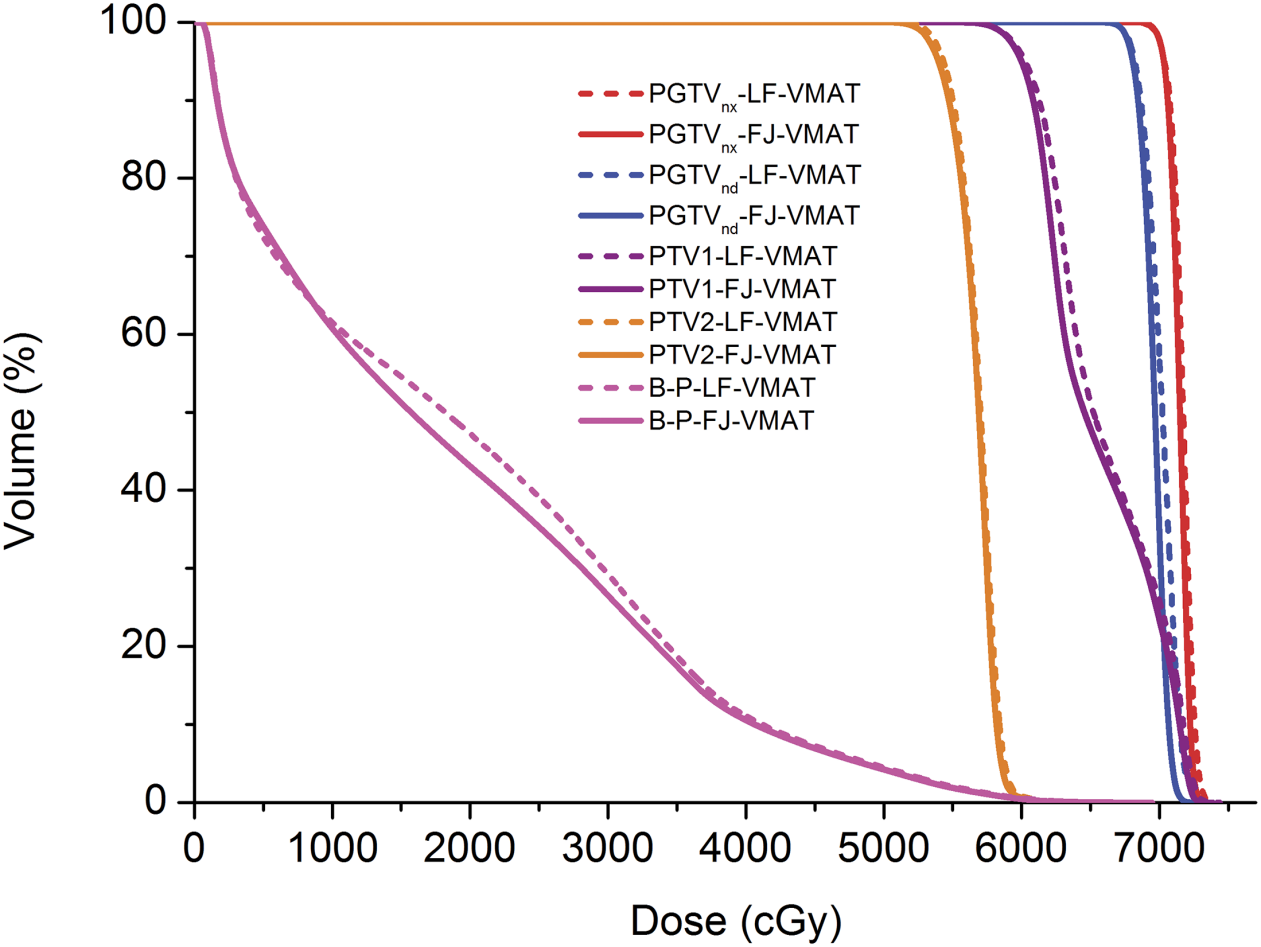
Dose-volume histograms (DVHs) of the large-field volumetric arc therapy (LF-VMAT) and fixed-jaw VMAT (FJ-VMAT) plans. The solid line: the FJ-VMAT plans. The dotted line: the LF-VMAT plans.

Table 2 summarizes the target coverage parameters between the two plans. Compared with the LF-VMAT plans, the FJ-VMAT plans provided comparable HI and CI (*P* > 0.05) of the PGTVnx and lower D_2%_ (by 1.6%, 1.0%, and 1.0%, respectively) of the PGTVnd, PTV1 and PTV2 (*P* < 0.05). The results are also illustrated in Figs 1 and 2.

**Table 2:** Dose-volume parameters of the targets for the large-field volumetric arc therapy (LF-VMAT) and fixed-jaw VMAT (FJ-VMAT) plans (mean ± SD).

Target	Parameter	LF-VMAT	FJ-VMAT	*P*-value
**PGTVnx**	**V**_**93%**_ **(%)**	100.00 ± 0.00	100.00 ± 0.00	1.000
	**D**_**mean**_ **(Gy)**	71.92 ± 0.32	71.71 ± 0.31	0.124
	**D**_**98%**_ **(Gy)**	69.89 ± 0.14	70.00 ± 0.27	0.231
	**D**_**50%**_ **(Gy)**	71.99 ± 0.36	71.76 ± 0.32	0.121
	**D**_**2%**_ **(Gy)**	73.43 ± 0.52	73.06 ± 0.57	0.055
	**CI**	0.84 ± 0.06	0.85 ± 0.07	0.539
	**HI**	0.05 ± 0.01	0.04 ± 0.01	0.221
**PGTVnd**	**V**_**93%**_ **(%)**	99.99 ± 0.03	99.99 ± 0.00	0.343
	**D**_**mean**_ **(Gy)**	70.48 ± 0.46	69.91 ± 0.39	0.000
	**D**_**98%**_ **(Gy)**	67.85 ± 0.51	67.76 ± 0.25	0.601
	**D**_**50%**_ **(Gy)**	70.49 ± 0.48	69.98 ± 0.40	0.001
	**D**_**2%**_ **(Gy)**	72.79 ± 1.16	71.61 ± 0.78	0.000
**PTV1**	**V**_**93%**_ **(%)**	99.91 ± 0.09	99.93 ± 0.13	0.601
	**D**_**mean**_ **(Gy)**	65.83 ± 0.77	65.38 ± 0.80	0.001
	**D**_**98%**_ **(Gy)**	59.04 ± 0.36	59.31 ± 0.46	0.089
	**D**_**50%**_ **(Gy)**	65.22 ± 0.95	64.46 ± 0.93	0.000
	**D**_**2%**_ **(Gy)**	72.89 ± 0.45	72.41 ± 0.54	0.003
**PTV2**	**V**_**93%**_ **(%)**	99.98 ± 0.04	99.97 ± 0.06	0.566
	**D**_**mean**_ **(Gy)**	57.08 ± 0.47	56.76 ± 0.49	0.012
	**D**_**98%**_ **(Gy)**	53.62 ± 0.44	53.44 ± 0.42	0.134
	**D**_**50%**_ **(Gy)**	57.16 ± 0.48	56.83 ± 0.54	0.019
	**D**_**2%**_ **(Gy)**	60.26 ± 1.56	59.94 ± 1.39	0.040

V_x_, volume receiving at least x dose; D_x%_, dose that is reached or exceeded in x% of the volume; D_mean_, mean dose; CI, conformity index; HI, homogeneity index; PTV, planning target volume; PGTVnx, PTV of primary nasopharynx; PGTVnd, PTV of positive lymph nodes; PTV1, PTV of high-risk region; PTV2, PTV of low-risk region.

### OAR sparing

Table 3 summarizes the dose-volume parameters of the OARs between the two plans. Compared with the LF-VMAT plans, the FJ-VMAT plans provided 1.8% lower D_max_ and 16.5% lower V_54Gy_ of PRV_Bstem_, 1.4% lower D_max_ of PRV_SC_, 4.1% lower D_mean_ and 9.8% lower V_30Gy_ of left parotid, 3.0% lower D_mean_ and 8.0% lower V_30Gy_ of right parotid, and 5.0% lower V_20Gy_ and 8% lower V_30Gy_ of B-P (*P* < 0.05). In terms of the dose delivered to the oral cavity and larynx, no significant differences were observed. The results are also illustrated in Figs 1 and 2. The isodose lines of the low-dose area of normal tissue were clustered more tightly around the target for the FJ-VMAT plans.

**Table 3:** Dose-volume parameters of the organs at risk (OARs) for the large-field volumetric arc therapy (LF-VMAT) and fixed-jaw VMAT (FJ-VMAT) plans (mean ± SD).

OAR	Parameter	LF-VMAT	FJ-VMAT	*P*-value
**PRV**_**Bstem**_	**D**_**max**_ **(Gy)**	63.13 ± 3.44	62.02 ± 4.12	0.007
	**V**_**54Gy**_ **(cc)**	1.15 ± 0.61	0.96 ± 0.70	0.039
	**V**_**60Gy**_ **(cc)**	0.18 ± 0.25	0.17 ± 0.26	0.618
**PRV**_**SC**_	**D**_**max**_ **(Gy)**	48.21 ± 2.36	47.52 ± 2.70	0.027
	**V**_**45Gy**_ **(cc)**	0.16 ± 0.20	0.12 ± 0.20	0.205
	**V**_**50Gy**_ **(cc)**	0.00 ± 0.00	0.01 ± 0.02	0.343
**Left parotid**	**D**_**mean**_ **(Gy)**	37.77 ± 1.49	36.22 ± 1.58	0.005
	**V**_**30Gy**_ **(%)**	61.16 ± 8.58	54.56 ± 4.62	0.013
**Right parotid**	**D**_**mean**_ **(Gy)**	37.94 ± 2.10	36.79 ± 2.43	0.015
	**V**_**30Gy**_ **(%)**	62.04 ± 11.26	56.98 ± 9.36	0.027
**Oral cavity**	**D**_**mean**_ **(Gy)**	39.44 ± 5.79	38.97 ± 5.26	0.117
**Larynx**	**D**_**mean**_ **(Gy)**	38.65 ± 2.30	39.01 ± 3.49	0.809
**B-P**	**V**_**5Gy**_ **(%)**	67.47 ± 11.44	67.13 ± 11.58	0.199
	**V**_**10Gy**_ **(%)**	55.28 ± 10.48	54.85 ± 10.58	0.109
	**V**_**20Gy**_ **(%)**	41.37 ± 8.56	39.33 ± 8.04	0.003
	**V**_**30Gy**_ **(%)**	27.98 ± 6.34	25.71 ± 5.76	0.001

V_x_, volume receiving at least x dose; D_x%_, dose that is reached or exceeded in x% of the volume; D_max_, maximum dose; D_mean_, mean dose; PRV, planning OAR volume; PRV_Bstem_, PRV of brainstem; PRV_SC_, PRV of spinal cord; B-P, region of body minus all planning target volumes (PTVs).

### Dose delivery parameter

As shown in Table 4, the MUs of the FJ-VMAT plans (683 ± 87) increased by 22% ± 12% compared with those of the LF-VMAT plans (559 ± 62). With respect to the TT, no statistical significance was observed.

**Table 4:** Monitor units and treatment time for the large-field volumetric arc therapy (LF-VMAT) and fixed-jaw VMAT (FJ-VMAT) plans (mean ± SD).

Parameter	LF-VMAT	FJ-VMAT	Difference	*P*-value
**Monitor units**	559 ± 62	683 ± 87	124 ± 68	0.000
**Treatment time (s)**	127.3 ± 0.7	127.5 ± 0.8	0.2 ± 0.6	0.343

## Discussion

Because many studies [1–3,11] have demonstrated that VMAT is superior or comparable to IMRT for NPC cases, we did not compare the FJ-VMAT with IMRT in this study. The primary aim of this study was to clarify the dosimetric benefits of the FJ-VMAT technique for NPC. Our results show that FJ-VMAT could achieve lower maximum and mean doses in terms of the PGTVnd, PTV1, PTV2 and could spare the Bstem, SC, parotid, and normal tissue compared to LF-VMAT. With a similar or slightly superior dose distribution of the targets, FJ-VMAT can reduce the doses delivered to OARs, especially to the low-dose regions of normal tissue (Fig 1). The results are similar to those reported in our previous study [8], which focused on cervical cancer. We found that FJ-VMAT could provide similar target coverage and superior OAR sparing compared with LF-VMAT, with much more tightly clustered lower isodose lines. However, the study by Srivastava et al. [7], which focused on the fixed-jaw technique applied in head and neck IMRT, showed that fixed-jaw IMRT was similar to split-jaw IMRT in terms of plan quality. The authors’ results were different from those obtained in our study because the split-jaw IMRT could provide sufficient MLC modulation but at the expense of increasing the split field number.

**Fig 3:**
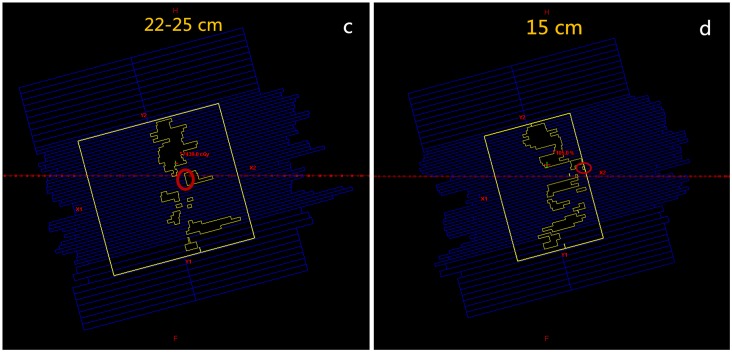
Jaw widths and ranges of multi-leaf collimator (MLC) motion in the large-field volumetric arc therapy (LF-VMAT) and fixed-jaw VMAT (FJ-VMAT) plans. (c) The LF-VMAT plans. (d) The FJ-VMAT plans. Circle: the mechanical limitation of an MLC whose maximum displacement is 15 cm.

The jaw position of the Varian linear accelerator is designed to remain unchanged when the MLC moves and shapes the aperture. When the VMAT technique is applied to a large target volume, such as the targets of NPC, which are typically larger than 20 cm, the mechanical leaf span limitation of 15 cm restricts delivery. In LF-VMAT, the jaw width is automatically set to > 15 cm. In this study, the MLC on one side of the field could not reach the regions on the other side along the X direction (Fig 3c). In contrast, the MLC in FJ-VMAT could move to any position in the field along the X direction with a high degree of freedom (Fig 3d).

In terms of OAR sparing, the MLC of LF-VMAT is not able to shield the OAR area which lies outside of the MLC leaf span, but the MLC of FJ-VMAT is able to shield the entire OAR area in the field. Moreover, the jaw of FJ-VMAT, which allows much less leakage than MLC, shields the OARs outside of the field [8].

In terms of target coverage, the MLC in FJ-VMAT can better modulate the beam intensity during the optimization process [8]; therefore, a similar or superior dose distribution of targets can be easier to achieve. Although the FJ-VMAT field only covers a portion of the target area at some gantry angles, the remaining target area can be covered at other gantry angles; therefore, more output MUs, approximately 22% ± 12% in this study, were needed to achieve a sufficient target dose. It is known that the scattered radiation administered to a patient’s body outside of the treatment volume is first-order directly proportional to the applied MUs in the treatment. Thus, in theory, the excessive number of MUs required by FJ-VMAT could increase the risk of radiation-induced secondary malignancies. With respect to the treatment time, no significant differences were observed because the main limitation affecting the treatment time was the gantry rotation, and the gantry rotations in both plans maintained a maximum speed of 6°/s in most of the control points; thus, the treatment times were equivalent. However, Srivastava et al. [7] concluded that fixed-jaw IMRT could reduce the number of MUs and the beam-on time. The author’s results are different from ours because the fixed-jaw technique can reduce the number of split fields automatically generated by the treatment planning system.

## Conclusion

In this study, we investigated the dosimetric benefits of the fixed-jaw technique applied in the VMAT of NPC, and we found that it can provide a similar or slightly superior target dose distribution and spare most of the OARs more effectively, with an increased number of MUs and equivalent treatment time. Thus, the FJ-VMAT technique is the preferred technique at our center.
